# The ED-PLANN Score: A Simple Risk Stratification Tool for Out-of-Hospital Cardiac Arrests Derived from Emergency Departments in Korea

**DOI:** 10.3390/jcm11010174

**Published:** 2021-12-29

**Authors:** Hyouk Jae Lim, Young Sun Ro, Ki Hong Kim, Jeong Ho Park, Ki Jeong Hong, Kyoung Jun Song, Sang Do Shin

**Affiliations:** 1 Department of Emergency Medicine, Seoul National University Hospital, Seoul 03080, Korea; cypress668@gmail.com (H.J.L.); emphysiciankkh@gmail.com (K.H.K.); timthe@gmail.com (J.H.P.); emkjhong@gmail.com (K.J.H.); sdshin@snu.ac.kr (S.D.S.); 2 Laboratory of Emergency Medical Services, Seoul National University Hospital Biomedical Research Institute, Seoul 03080, Korea; skciva@gmail.com; 3 Department of Emergency Medicine, Seoul National University Boramae Medical Center, Seoul 07061, Korea

**Keywords:** cardiac arrest, big data, prediction model, prognosis

## Abstract

Early risk stratification of out-of-hospital cardiac arrest (OHCA) patients with insufficient information in emergency departments (ED) is difficult but critical in improving intensive care resource allocation. This study aimed to develop a simple risk stratification score using initial information in the ED. Adult patients who had OHCA with medical etiology from 2016 to 2020 were enrolled from the Korean Cardiac Arrest Research Consortium (KoCARC) database. To develop a scoring system, a backward logistic regression analysis was conducted. The developed scoring system was validated in both external dataset and internal bootstrap resampling. A total of 8240 patients were analyzed, including 4712 in the development cohort and 3528 in the external validation cohort. An ED-PLANN score (range 0–5) was developed incorporating 1 point for each: P for serum pH ≤ 7.1, L for serum lactate ≥ 10 mmol/L, A for age ≥ 70 years old, N for non-shockable rhythm, and N for no-prehospital return of spontaneous circulation. The area under the receiver operating characteristics curve (AUROC) for favorable neurological outcome was 0.93 (95% CI, 0.92–0.94) in the development cohort, 0.94 (95% CI, 0.92–0.95) in the validation cohort. Hosmer–Lemeshow goodness-of-fit tests also indicated good agreement. The ED-PLANN score is a practical and easily applicable clinical scoring system for predicting favorable neurological outcomes of OHCA patients.

## 1. Introduction

Out-of-hospital cardiac arrest (OHCA) is a major public health burden due to its low survival rate, along with neurological and functional disability [1]. An aging population with increasing comorbidities has increased the incidence of OHCA, despite advances in resuscitation improving the global survival rate over the past few decades, and the survival outcomes after cardiac arrest remain low [2]. Several highly resource-intensive treatments, including extracorporeal membrane oxygenation, percutaneous coronary intervention, and targeted temperature management, have the potential to improve outcomes for OHCA patients [3,4,5,6]. Since the probability of survival decreases rapidly after cardiac arrest, the highly resource-intensive treatments should be started immediately when indicated [7,8]. Early risk stratification of OHCA patients with insufficient information for prognosis in emergency settings is difficult but critical to improving intensive resource allocation, preventing costly investigations, and informing family members for discussion [9,10]. However, the level of existing risk stratification tools is limited in predicting probabilities of survival and determining the indications for intensive care immediately after OHCA patients arrive at the emergency department (ED).

Information attainable initially in the ED, including patients’ characteristics, prehospital factors, and biochemical variables from Point-of-Care Test (POCT), was associated with clinical outcomes after OHCA [11,12,13,14,15,16,17]. Risk stratification tools consisting only of a combination of patients’ demographics and prehospital factors that can provide a simple and rapid assessment are generally used for termination of resuscitation rules at the scene [18,19]. Other risk scoring systems predict clinical outcomes of OHCA based on data that are not initially obtainable or difficult to calculate in the ED, potentially limiting routine and timely use of these tools in emergencies [20,21,22]. Therefore, there is an unmet need for a simple clinical scoring system that can be applied within the first few minutes after an OHCA patient arrives at the ED and that can predict survival outcomes with high sensitivity and specificity.

We hypothesized that prognostic predictors based on a set of information attainable initially in the ED could provide risk stratification of OHCA survival outcomes with high accuracy and support clinical decision making in emergencies. The purposes of this study were to develop a simple risk stratification score using initial information in the ED and to validate the risk stratification score by verifying predictive performance and clinical usefulness.

## 2. Materials and Methods

### 2.1. Study Design, Setting, and Data Sources

This study was a population-based observational study using the Korean Cardiac Arrest Research Consortium (KoCARC) database. The KoCARC is a nationwide multicenter network for OHCA data collection and collaborative research across Korea. The KoCARC investigators have been prospectively collecting predetermined data from OHCA patients at the level 1 EDs of 32 university teaching hospitals since October 2015. The KoCARC registry included patients of OHCA transported to the participating ED by emergency medical services (EMS) with resuscitation efforts and patients who had a medical etiology identified by emergency physicians in each ED.

The Korean EMS system is a government-operated and two-tiered system that offers basic to intermediate levels of life support ambulance services from fire stations. A dual dispatch response system has been implemented for suspected OHCA cases since 2015. All EMS providers provide basic life support (BLS), and EMS providers with the qualifications of level I emergency medical technicians and nurses can apply advanced airways, such as endotracheal intubation, and can provide intravenous fluid to a patient following the EMS cardiopulmonary resuscitation (CPR) protocol. In the protocol for on-scene CPR, it is recommended that at least 3 cycles of resuscitative efforts should be delivered before transporting the patient to the hospital. Since only doctors can declare death in Korea, EMS providers transport all OHCA patients to an ED, continuing CPR except in cases where there are obvious signs of death, such as lividity and/or rigor mortis [23].

The KoCARC registry excluded OHCA patients with terminal illnesses documented by medical records, patients under hospice care, pregnant patients, and patients with a previously documented ‘Do Not Resuscitate’ card. OHCA patients of definite non-medical etiology, including trauma, drowning, poisoning, burn, asphyxia, or hanging, were also excluded. The registry was constructed using EMS run-sheets, EMS cardiac arrest registry, dispatcher CPR registry, and medical record reviews for hospital care and outcomes, which are extracted by the medical record reviewers. The data were collected following standardized Utstein-style templates for OHCA to facilitate uniform reporting using precisely defined variables and outcomes. The quality management committee consisting of emergency physicians, statistical experts, local research coordinators, and investigators in each ED provided feedback regarding quality management processes to the research coordinators and investigators. A detailed description of the KoCARC database was published in previous papers [24].

### 2.2. Study Population

This study included all adult EMS-treated OHCA patients with presumed medical etiology. The study period was from January 2016 to October 2020. Exclusion criteria were patients younger than 18 years of age, those who did not receive resuscitation in the ED due to immediate declaration of death by an emergency physician, or those whose components of initial blood gas analysis were missing.

For the development cohort, OHCA patients who had an initial blood gas analysis were included in the analysis. For the validation cohort, two separate samples were used. In the first one for external validation, OHCA patients with missing values for at least one component in laboratory data were included. In the second one for internal validation, bootstrap with 10,000 resamples from the development cohort were included.

### 2.3. Outcome Measures

The primary outcome was favorable neurological outcome, and the secondary outcome was survival to hospital discharge. Neurological outcome was evaluated according to the cerebral performance categories (CPC) scale [25], and CPC 1 and 2 were classified as favorable neurological outcomes.

### 2.4. Measurements and Variables

The main exposure variable of this study was prognostic predictors based on the set of information initially available in the ED. We investigated all potential prognostic predictors including patients’ demographics (age, gender, past medical history (diabetes mellitus, hypertension, and dyslipidemia), residential area (metropolitan or urban/rural), and place of cardiac arrest), prehospital factors (witness status, bystander CPR, bystander defibrillation, initial electrocardiogram (ECG) at the scene, EMS time variables (response time interval, scene time interval, and transport time interval), EMS defibrillation, mechanical CPR, epinephrine, prehospital return of spontaneous circulation (ROSC)), and initial POCT for blood gas analysis after arrival at the ED.

In cases where subsequent conversion from initially non-shockable rhythm to a shockable one was observed, they were included into the initially non-shockable rhythm group. If re-arrest occurred after achieving prehospital ROSC, it was considered as the prehospital ROSC group.

Immediately after the OHCA patients were admitted to the ED, blood collection was performed as soon as possible and blood gas analysis result including pH, pCO2, pO2, and lactate were reported within a few minutes using a commercially available POCT analyzer. All KoCARC participating hospitals were contributing to qualification programs such as the College of American Pathologists survey.

### 2.5. Statistical Analysis

In the development cohort, variables for patient demographics, prehospital factors, and initial POCT blood gas analysis were tested for association with favorable neurological outcomes. All variables satisfying *p* < 0.02 in univariate analysis were included in a backward stepwise logistic regression analysis to develop the final model. 

For model development, continuous variables including age, pH, pCO2, pO2, and lactate, were converted to categorical variables using the area under the receiver operating characteristics curve (AUROC). Cutoff values for predicting favorable neurological outcomes were calculated. Categorical variables were purposefully used to develop a simple scoring system that could be easily applied in an ED setting. To evaluate the performance of the scoring system, Hosmer–Lemeshow goodness-of-fit tests were performed to evaluate the calibration [26]. All variables included in the final model were assessed for multicollinearity. No significant collinearity was detected.

In the validation cohort for external validation, the model was applied to all patients and AUROC was calculated. Multiple imputations using multivariable proportional logistic regression models were conducted for missing components in blood gas analysis of the validation cohort. The Hosmer–Lemeshow goodness-of-fit tests were also performed in the validation cohort for calibration.

A bootstrap with 10,000 resamples from the development cohort was used for internal validation of the scoring system. It is well known that bootstrap methods are very efficient because the entire dataset is used for model development, and there is no need to collect new data for validation. Moreover, bootstrapping has been known to provide nearly unbiased estimates of prediction accuracy with relatively low variance [27]. To evaluate the scoring system in the bootstrap resample group, an AUROC greater than 0.80 was considered as indicative of good discrimination [28].

Sensitivity analysis was performed using the beta coefficient of each variable associated with a poor outcome. Schneeweiss’s beta scoring weight increases by 1 unit for each 0.3 increase in the beta, which refers to an e^0.30 = 35% increase in outcomes, where e is the mathematical constant 2.7182 [29]. This Schneeweiss’s beta weight was applied to the model and translated into a wide range of scores.

All statistical analysis was performed using SAS software, version 9.4 (SAS Institute Inc., Cary, NC, USA).

## 3. Results

**Figure 1:**
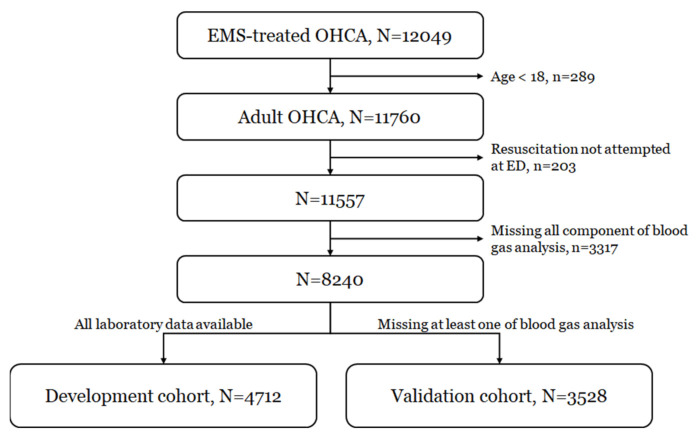
Study flow chart of the development and validation cohorts. OHCA: out-of-hospital cardiac arrest, EMS: emergency medical services, ED: emergency department.

Of the 12,049 EMS-treated OHCA patients during the study period, a total of 8240 patients were analyzed, excluding patients who were younger than 18 years of age (n = 289), those who did not receive resuscitation in ED due to immediate declaration of death (n = 203), and patients without data for all component of blood gas analysis (n = 3317). Supplementary Table S1 shows the characteristics of patients who were excluded (n = 3317) due to missing values for blood gas analysis. Among them, 4712 patients were in the development cohort and 3528 patients were in the external validation cohort. The bootstrap samples from the development cohort were included in the internal validation cohort. (Figure 1).

Demographic characteristics according to neurological outcomes of the development cohort patients are described in Table 1. Of the 4712 eligible patients, 603 (12.8%) of development cohort patients had favorable neurological outcomes. The favorable outcome group was younger than the poor outcome group (median (interquartile range, IQR) of age: 57 (49–67) vs. 73 (60–81), *p* < 0.01). Serum lactate levels were significantly lower (7.5 mmol/L vs. 12.4 mmol/L, *p* < 0.01), and the serum pH levels were significantly higher (7.26 vs. 6.91, *p* < 0.01) in the favorable outcome group compared with the poor outcome group. Initial shockable rhythm at the scene and prehospital ROSC were more likely in the favorable outcome group. Demographic characteristics of the external validation cohort according to neurological outcome are described in Supplementary Table S2. The proportion of patients living in metropolitan cities was smaller in the external validation cohort compared with the development cohort. Supplementary Table S3 shows the distribution of blood gas analysis components before and after multiple imputation for the external validation cohort.

For the scoring system development, a backward stepwise logistic regression analysis was conducted, and age, initial shockable ECG rhythm, prehospital ROSC, serum pH, and serum lactate were selected. Continuous variables were transformed into categorical variables using AUROC for predicting neurological outcomes, as described above. The cutoff values were 70 years old for age, 7.1 for initial serum pH, and 10 mmol/L for serum lactate.

Beta coefficients and odds ratios for retained variables in the multivariable logistic regression model predicting favorable neurologic outcomes can be found in Table 2. For simplicity of application, a scoring system ranging from 0 to 5 points was created, giving each variable an equal weight (1 point), as preplanned. Abbreviations were used for the ED-PLANN score: P for serum pH ≤ 7.1, L for serum lactate ≥10 mmol/L, A for age ≥70, N for non-shockable rhythm, and N for no-prehospital ROSC.

The ED-PLANN score = (if pH ≤ 7.1) + (if lactate ≥ 10 mmol/L) + (if age ≥ 70 years-old) + (if non-shockable rhythm) + (if no prehospital ROSC)

Schneeweiss beta scoring was applied in the sensitivity analysis for the wide range scoring system, with the modified ED-PLANN score ranging from 0 to 12.

The modified ED-PLANN score = 2 (if pH ≤ 7.1) + (if lactate ≥ 10 mmol/L) + 2 (if age ≥ 70 years old) + 3 (if non-shockable rhythm) + 4 (if no prehospital ROSC)

For converting the ED-PLANN score to the probability of a favorable neurological outcome, the following equations were determined.

Logit = 1.7833 − (1.4496 × ED-PLANN score)

**Figure 2:**
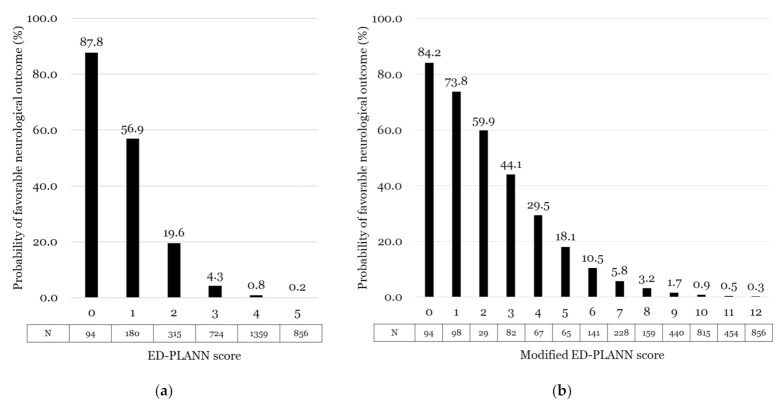
Probability of favorable neurological outcome in the validation cohort: (**a**) probability of favorable neurological outcome of the ED-PLANN score; (**b**) probability of favorable neurological outcome of the modified ED-PLANN score.

The equation for probability of a favorable neurological outcome = $e^{Logit}$/(1 + $e^{Logit}$) was formed. For converting the modified ED-PLANN score to the probability of a favorable neurological outcome, the same approach as described above was applied. (Hosmer–Lemeshow goodness-of-fit tests: statistic = 7.702 and *p* = 0.053 for the ED-PLANN score and statistic = 12.862 and *p* = 0.003 for the modified ED-PLANN score in the development cohort). Figure 2 and Supplementary Figure S1 show the probability of a favorable neurological outcome and survival to discharge of the ED-PLANN score and the modified ED-PLANN score for the validation cohort.

Table 3 shows AUROC for survival outcomes of the scores in the development and validation cohorts. The AUROC (95% CI) of the ED-PLANN score in the development cohort was 0.93 (0.92–0.94) for favorable neurological outcome and 0.88 (0.86–0.89) for survival to discharge. The AUROC of the internal validation cohort was 0.93 (0.92–0.93) for favorable neurological outcome and 0.87 (0.86–0.88) for survival to discharge, demonstrating excellent accuracy. The AUROC of the external validation cohort was 0.94 (0.92–0.95) for favorable neurological outcome and 0.85 (0.83–0.87) for survival to discharge. The values of the AUROC (95% CI) of the modified ED-PLANN score were similar with those of the ED-PLANN score. The Hosmer–Lemeshow statistics for favorable neurological outcome of the external validation cohort were 4.408 (*p* = 0.221) for the ED-PLANN score and 7.06 (*p* = 0.216) for the modified ED-PLANN score. 

## 4. Discussion

A simple scoring system was developed and validated based on a set of information initially available in the ED: the ED-PLANN score (range 0–5), with P for serum pH ≤ 7.1, A for age ≥ 70 years old, L for serum lactate ≥ 10 mmol/L, N for non-shockable rhythm, and N for no-prehospital ROSC. The ED-PLANN score and the modified ED-PLANN score predict chances of survival with good neurological outcome with high accuracy (both, AUROC 0.93 (0.92–0.94)) and with good agreement of calibration. Early and accurate prediction using the ED-PLANN score could support rapid decision-making in emergency settings to achieve a better prognosis of time-sensitive OHCA outcomes.

Currently available risk scoring systems for predicting survival outcomes of OHCA have limitations when applied in emergency settings. In the early development of the cardiac arrest scoring system, the OHCA score, proposed in France, included parameters that would be available after admission to the intensive care unit (ICU) [20]. The OHCA score integrates biochemical variables at the time of ICU admission. Some variables such as no-flow time and low-flow time are complex, and the score calculation is based on a complex weighting system and the use of natural logarithm. Another scoring system, the CAHP score, an abbreviation for Cardiac Arrest Hospital Prognosis score, can be calculated using a nomogram consisting of age, location of arrest, initial ECG rhythm, duration from the initial collapse to BLS, duration from BLS to ROSC, pH, and epinephrine dose [22]. The CAHP score is difficult to apply in emergency settings and requires data that are not easily available in the ED, such as minutes to start of CPR or defibrillation [30,31]. In this study, the ED-PLANN score was developed based on information of age, initial rhythm, prehospital ROSC status, and POCT blood gas analysis, which are initially obtainable in the ED. Therefore, this score can provide reliable and practical predictive probabilities for OHCA patients’ outcomes within a few minutes of arriving at the ED.

Despite the unsolved challenging ethical issues that may be involved, early and accurate prognostic assessment may be useful for medical teams to help in making decisions and in allocating resources to those most likely to benefit from them. Early and accurate prognostic assessment is necessary, especially in the ED, as the prognosis is known to decrease rapidly during CPR without ROSC, and highly resource-intensive treatments such as mechanical circulatory support should be started immediately when indicated [7,8]. The ED-PLANN score can provide an early and accurate prognosis using variables that can be easily obtained with simple calculations. However, the convenience of simple scoring can miss a treatable cause of cardiac arrest. Physicians should decide to stop resuscitation efforts by gathering additional critical information and repeatedly examining the patient’s condition.

There was a sharp drop in favorable probabilities between the ED-PLANN score of 2 and 3. However, the ED-PLANN score only provides the probabilities of clinical outcomes, and this sharp drop does not imply a cutoff value. In the sensitivity analysis, a wide range scoring system was developed, the modified ED-PLANN score, to provide a wide range of scores for these borderline zones, and this could support difficult but crucial decision making for emergency clinicians. However, in the aspect of simplicity and usability, the ED-PLANN may be more useful than the modified ED-PLANN score.

The ED-PLANN scoring system consists of information obtained at the scene, with the exception of serum lactate level. If a prehospital POCT analyzer is available at the CPR scene and EMS providers perform advanced cardiac life support (ACLS), the ED-PLANN score will be applicable in a prehospital setting, enabling the selection of an appropriate hospital for intensive treatment or supporting a decision of termination of resuscitation. For countries with a BLS level of an EMS system, the ED-PLANN score may be applied in early ED or intensive care unit settings. In many environments with different EMS systems, this simple ED-PLANN score will support early decision-making for OHCA. Further studies are needed to confirm the usefulness of the ED-PLANN score in various EMS systems and prehospital settings.

### Limitations 

This study has several limitations. First, this study was conducted in a two-tiered, dual dispatch EMS system providing basic to intermediate service level, which is different from other countries. Noteworthy differences were also found in the OHCA patients’ characteristics and proportion of field termination of resuscitation of the other registries. These factors could have influenced study outcomes, limiting the generalizability of the findings of this study. The organization of EMS varies across countries, and the ED-PLANN score may need to be calibrated and validated for each specific system to obtain generalizability. Second, as this study was an observational study, there might have been potential biases that were not controlled. In this study, physicians were not blinded to the prognostic variables such as age, initial shockable rhythm, prehospital ROSC status, or POCT blood gas analysis, which could influence physicians’ clinical decision making and patients’ prognosis. To make an evaluation of prognostic variables without biases, physicians should be blinded to the prognostic variables so as not to influence their resuscitation efforts and decisions. The potential for impact due to these bias issues is significant; therefore, the study findings should be interpreted with caution. Third, this study registry had no distinction in whether blood samples were taken from an artery or a vein. Blood sampling is technically difficult, especially during CPR. Differences in the results of arterial and venous blood gas analyses taken during CPR have been reported, even though the decision of a physician based on blood sampling is little changed [32]. Fourth, there were missing values in the blood gas analysis in the external validation cohort. The major concerns of POCT analysis were imprecision, performance interferences, and missing values compared with routine laboratory analysis [33]. These intrinsic characteristics of POCT values might have been potential biases that were not controlled. Fifth, this simple scoring system was developed based on a set of information attainable initially in the ED. Some crucial prognostic predictors, such as estimated time of cardiac arrest and time intervals from arrest to EMS arrival at the scene, were not included in the model because they might be difficult to obtain from bystanders who witnessed the arrest in emergency settings. Lastly, the early risk stratification of survival outcomes would only be needed for OHCA patients without alert mentality at the time of ED arrival. However, information on the mental status at the time of ED arrival is not available in the KoCARC database. Among 8240 eligible patients of this study, 266 (3.2%) patients with prehospital ROSC had a GCS score of 14 or 15 at the time of hospital admission. Therefore, it can act as a potential bias.

## 5. Conclusions

The ED-PLANN score is a practical and easily adaptable clinical scoring system for predicting favorable neurological outcomes and survival to discharge of OHCA patients, with high accuracy within minutes of ED arrival. Using a simple method for predicting the prognosis of OHCA patients in the ED early would help physicians make early treatment planning decisions, thus providing a better prognosis for OHCA patients.

## Figures and Tables

**Table 1:** Characteristics of the development cohort population.

Development Cohort		Total	Favorable Neurological Outcome	Poor Neurological Outcome	*p*–Value
		N (%)	N (%)	N (%)	
Total		4712	603	4109	
Sex, female		3145 (66.7)	470 (77.9)	2675 (65.1)	<0.01
Age, years					<0.01
	18–49	613 (13.0)	155 (25.7)	458 (11.1)	
	50–59	747 (15.9)	194 (32.2)	553 (13.5)	
	60–69	878 (18.6)	147 (24.4)	731 (17.8)	
	70–79	1242 (26.4)	75 (12.4)	1167 (28.4)	
	80–120	1232 (26.1)	32 (5.3)	1200 (29.2)	
	Median (IQR)	71 (58–80)	57 (49–67)	73 (60–81)	<0.01
Past medical history					
	Diabetes mellitus	1224 (26.0)	109 (18.1)	1115 (27.1)	<0.01
	Hypertension	1898 (40.3)	229 (38.0)	1669 (40.6)	0.22
	Dyslipidemia	238 (5.1)	42 (7.0)	196 (4.8)	0.02
Residence of patient					0.04
	Metropolitan	3185 (67.6)	386 (64.0)	2799 (68.1)	
Place of arrest					<0.01
	Public	1273 (27.0)	255 (42.3)	1018 (24.8)	
	Private	2763 (58.6)	232 (38.5)	2531 (61.6)	
	Others	676 (14.3)	116 (19.2)	560 (13.6)	
Witnessed		2993 (63.5)	510 (84.6)	2483 (60.4)	<0.01
Bystander CPR		2474 (52.5)	359 (59.5)	2115 (51.5)	<0.01
Bystander defibrillation		39 (0.8)	21 (3.5)	18 (0.4)	<0.01
Initial shockable rhythm at the scene		981 (20.8)	437 (72.5)	544 (13.2)	<0.01
EMS time, minute					
	Response time interval, median (IQR)	7 (5–10)	7 (5–9)	7 (5–10)	<0.01
	Scene time interval, median (IQR)	13 (9–17)	10 (6–14)	13 (9–18)	<0.01
	Transport time interval, median (IQR)	9 (6–13)	11 (7–17)	9 (6–13)	<0.01
Prehospital treatment					
	EMS defibrillation	1228 (26.1)	461 (76.5)	767 (18.7)	<0.01
	Mechanical CPR device	1000 (21.2)	33 (5.5)	967 (23.5)	<0.01
	Epinephrine	777 (16.5)	44 (7.3)	733 (17.8)	<0.01
Prehospital ROSC		860 (18.3)	480 (79.6)	380 (9.2)	<0.01
Initial blood gas analysis					
	pH, median (IQR)	6.93 (6.80–7.09)	7.26 (7.14–7.33)	6.91 (6.80–7.03)	<0.01
	pCO2, median (IQR)	70.0 (46.3–94.0)	37.1 (30.8–46.0)	74.7 (53.8–97.9)	<0.01
	pO2, median (IQR)	51.3 (24.0–86.5)	95.3 (65.5–163.1)	45.3 (22.6–76.8)	<0.01
	lactate, median (IQR)	11.8 (8.3–15.0)	7.5 (4.8–10.6)	12.4 (9.0–15.0)	<0.01
Post-resuscitation care					
	TTM	531 (11.3)	180 (29.9)	351 (8.5)	<0.01
	Reperfusion therapy	749 (15.9)	422 (70.0)	327 (8.0)	<0.01
	ECMO	141 (3.0)	30 (5.0)	111 (2.7)	<0.01
Survival to discharge		854 (18.1)	-	-	
Favorable neurological outcome		603 (12.8)	-	-	

Abbreviations: IQR, interquartile range; CPR, cardiopulmonary resuscitation; EMS, emergency medical services; ROSC, return of spontaneous circulation; TTM, targeted temperature management; ECMO, extracorporeal membrane oxygenation.

**Table 2:** ED-PLANN score and modified ED-PLANN score: simple risk stratification scores predicting favorable neurological outcomes for OHCA patients.

Abbreviations	Variables	Odds Ratio	95% CI	Beta Coefficient	Schneeweiss’s Beta Scoring	ED-PLANN Scoring	Modified ED-PLANN Scoring
P	pH ≤7.1	4.14	3.09–5.54	0.71	2	1	2
L	Lactate ≥10 mmol/L	1.69	1.27–2.24	0.26	1	1	1
A	Age ≥70 years	4.56	3.54–5.89	0.76	2	1	2
N	Non–shockable rhythm	4.56	3.54–5.89	0.76	3	1	3
N	No prehospital ROSC	9.11	6.98–11.89	1.10	4	1	4

Abbreviations: CI, confidence interval.

**Table 3:** Area under the receiver operating characteristic curve in the development and validation cohort groups.

		ED-PLANN Score		Modified ED-PLANN Score	
		AUROC	CI	AUROC	CI
Development cohort	Favorable neurological outcome	0.93	0.92–0.94	0.93	0.92–0.94
	Survival to discharge	0.88	0.86–0.89	0.88	0.87–0.90
Internal validation cohort	Favorable neurological outcome	0.93	0.92–0.93	0.93	0.92–0.94
	Survival to discharge	0.87	0.86–0.88	0.87	0.86–0.88
External validation cohort	Favorable neurological outcome	0.94	0.92–0.95	0.95	0.93–0.96
	Survival to discharge	0.85	0.83–0.87	0.86	0.84–0.88

## Data Availability

The data presented in this study are available on request from the corresponding author. The data are not publicly available because the distribution of data is determined after the KoCARC research committee deliberation.

## Supplementary Materials

The following are available online at https://www.mdpi.com/article/10.3390/jcm11010174/s1, Figure S1: Probability of survival to discharge in validation cohort: (a) probability of survival to discharge of the ED-PLANN score; (b) probability of survival to discharge of the modified ED-PLANN score, Table S1: Characteristics of excluded patients due to missing values for blood gas analysis, Table S2: Characteristics of external validation cohort population, Table S3: Comparison of blood gas analysis components between before and after multiple imputation for the external validation cohort.

## References

[1] Virani S.S., Alonso A., Aparicio H.J., Benjamin E.J., Bittencourt M.S., Callaway C.W., Carson A.P., Chamberlain A.M., Cheng S., Delling F.N. (2021). Heart Disease and Stroke Statistics-2021 Update: A Report from the American Heart Association. Circulation.

[2] Yan S., Gan Y., Jiang N., Wang R., Chen Y., Luo Z., Zong Q., Chen S., Lv C. (2020). The global survival rate among adult out-of-hospital cardiac arrest patients who received cardiopulmonary resuscitation: A systematic review and meta-analysis. Crit. Care.

[3] Kim S.J., Kim H.J., Lee H.Y., Ahn H.S., Lee S.W. (2016). Comparing extracorporeal cardiopulmonary resuscitation with conventional cardiopulmonary resuscitation: A meta-analysis. Resuscitation.

[4] Park J.H., Song K.J., Shin S.D., Ro Y.S., Hong K.J. (2019). Time from arrest to extracorporeal cardiopulmonary resuscitation and survival after out-of-hospital cardiac arrest. Emerg. Med. Australas..

[5] Spaulding C.M., Joly L.M., Rosenberg A., Monchi M., Weber S.N., Dhainaut J.F., Carli P. (1997). Immediate coronary angiography in survivors of out-of-hospital cardiac arrest. N. Engl. J. Med..

[6] Yannopoulos D., Bartos J.A., Aufderheide T.P., Callaway C.W., Deo R., Garcia S., Halperin H.R., Kern K.B., Kudenchuk P.J., Neumar R.W. (2019). The Evolving Role of the Cardiac Catheterization Laboratory in the Management of Patients with Out-of-Hospital Cardiac Arrest: A Scientific Statement from the American Heart Association. Circulation.

[7] Nagao K., Nonogi H., Yonemoto N., Gaieski D.F., Ito N., Takayama M., Shirai S., Furuya S., Tani S., Kimura T. (2016). Duration of Prehospital Resuscitation Efforts After Out-of-Hospital Cardiac Arrest. Circulation.

[8] Wengenmayer T., Rombach S., Ramshorn F., Biever P., Bode C., Duerschmied D., Staudacher D.L. (2017). Influence of low-flow time on survival after extracorporeal cardiopulmonary resuscitation (eCPR). Crit. Care.

[9] Martinell L., Nielsen N., Herlitz J., Karlsson T., Horn J., Wise M.P., Undén J., Rylander C. (2017). Early predictors of poor outcome after out-of-hospital cardiac arrest. Crit. Care.

[10] Pareek N., Kordis P., Beckley-Hoelscher N., Pimenta D., Kocjancic S.T., Jazbec A., Nevett J., Fothergill R., Kalra S., Lockie T. (2020). A practical risk score for early prediction of neurological outcome after out-of-hospital cardiac arrest: MIRACLE2. Eur. Heart J..

[11] Ho A.F.W., Hao Y., Pek P.P., Shahidah N., Yap S., Ng Y.Y., Wong K.D., Lee E.J., Khruekarnchana P., Wah W. (2019). Outcomes and modifiable resuscitative characteristics amongst pan-Asian out-of-hospital cardiac arrest occurring at night. Medicine.

[12] Huang J.B., Lee K.H., Ho Y.N., Tsai M.T., Wu W.T., Cheng F.J. (2021). Association between prehospital prognostic factors on out-of-hospital cardiac arrest in different age groups. BMC Emerg. Med..

[13] Lai C.Y., Lin F.H., Chu H., Ku C.H., Tsai S.H., Chung C.H., Chien W.-C., Wu C.-H., Chu C.-M., Chang C.-W. (2018). Survival factors of hospitalized out-of-hospital cardiac arrest patients in Taiwan: A retrospective study. PLoS ONE.

[14] Sasson C., Rogers M.A., Dahl J., Kellermann A.L. (2010). Predictors of survival from out-of-hospital cardiac arrest: A systematic review and meta-analysis. Circ. Cardiovasc. Qual Outcomes.

[15] Kim Y.J., Lee Y.J., Ryoo S.M., Sohn C.H., Ahn S., Seo D.W., Lim K.S., Kim W.Y. (2016). Role of blood gas analysis during cardiopulmonary resuscitation in out-of-hospital cardiac arrest patients. Medicine.

[16] Momiyama Y., Yamada W., Miyata K., Miura K., Fukuda T., Fuse J., Fuse J., Kikuno T. (2017). Prognostic values of blood pH and lactate levels in patients resuscitated from out-of-hospital cardiac arrest. Acute Med. Surg..

[17] Shinozaki K., Oda S., Sadahiro T., Nakamura M., Hirayama Y., Watanabe E., Tateishi Y., Nakanishi K., Kitamura N., Sato Y. (2011). Blood ammonia and lactate levels on hospital arrival as a predictive biomarker in patients with out-of-hospital cardiac arrest. Resuscitation.

[18] Morrison L.J., Verbeek P.R., Zhan C., Kiss A., Allan K.S. (2009). Validation of a universal prehospital termination of resuscitation clinical prediction rule for advanced and basic life support providers. Resuscitation.

[19] Morrison L.J., Visentin L.M., Kiss A., Theriault R., Eby D., Vermeulen M., Sherbino J., Verbeek P.R. (2006). Validation of a rule for termination of resuscitation in out-of-hospital cardiac arrest. N. Engl. J. Med..

[20] Adrie C., Cariou A., Mourvillier B., Laurent I., Dabbane H., Hantala F., Rhaoui A., Thuong M., Monchi M. (2006). Predicting survival with good neurological recovery at hospital admission after successful resuscitation of out-of-hospital cardiac arrest: The OHCA score. Eur. Heart J..

[21] Kiehl E.L., Parker A.M., Matar R.M., Gottbrecht M.F., Johansen M.C., Adams M.P., Griffiths L.A., Dunn S.P., Bidwell K.L., Menon V. (2017). C-GRApH: A Validated Scoring System for Early Stratification of Neurologic Outcome After Out-of-Hospital Cardiac Arrest Treated with Targeted Temperature Management. J. Am. Heart Assoc..

[22] Maupain C., Bougouin W., Lamhaut L., Deye N., Diehl J.L., Geri G., Perier M.C., Beganton F., Marijon E., Jouven X. (2016). The CAHP (Cardiac Arrest Hospital Prognosis) score: A tool for risk stratification after out-of-hospital cardiac arrest. Eur. Heart J..

[23] Kim K.H., Ro Y.S., Park J.H., Kim T.H., Jeong J., Hong K.J., Song K.J., Shin S.D. (2021). Association between case volume of ambulance stations and clinical outcomes of out-of-hospital cardiac arrest: A nationwide multilevel analysis. Resuscitation.

[24] Kim J.Y., Hwang S.O., Shin S.D., Yang H.J., Chung S.P., Lee S.W., Song K.J., Hwang S.S., Cho G.C., Moon S.W. (2018). Korean Cardiac Arrest Research Consortium (KoCARC): Rationale, development, and implementation. Clin. Exp. Emerg. Med..

[25] Safar P., Bleyaert A., Nemoto E.M., Moossy J., Snyder J.V. (1978). Resuscitation after global brain ischemia-anoxia. Crit. Care Med..

[26] Kramer A.A., Zimmerman J.E. (2007). Assessing the calibration of mortality benchmarks in critical care: The Hosmer-Lemeshow test revisited. Crit. Care Med..

[27] Steyerberg E.W., Harrell F.E., Borsboom G.J., Eijkemans M.J., Vergouwe Y., Habbema J.D. (2001). Internal validation of predictive models: Efficiency of some procedures for logistic regression analysis. J. Clin. Epidemiol..

[28] Hanley J.A., McNeil B.J. (1982). The meaning and use of the area under a receiver operating characteristic (ROC) curve. Radiology.

[29] Mehta H.B., Mehta V., Girman C.J., Adhikari D., Johnson M.L. (2016). Regression coefficient-based scoring system should be used to assign weights to the risk index. J. Clin. Epidemiol..

[30] Aschauer S., Dorffner G., Sterz F., Erdogmus A., Laggner A. (2014). A prediction tool for initial out-of-hospital cardiac arrest survivors. Resuscitation.

[31] Larsen M.P., Eisenberg M.S., Cummins R.O., Hallstrom A.P. (1993). Predicting survival from out-of-hospital cardiac arrest: A graphic model. Ann. Emerg. Med..

[32] Steedman D.J., Robertson C.E. (1992). Acid base changes in arterial and central venous blood during cardiopulmonary resuscitation. Arch Emerg. Med..

[33] Kapoor D., Srivastava M., Singh P. (2014). Point of care blood gases with electrolytes and lactates in adult emergencies. Int. J. Crit. Illn. Inj. Sci..

