# Supplementary material for: The ED-PLANN Score: A Simple Risk Stratification Tool for Out-of-Hospital Cardiac Arrests Derived from Emergency Departments in Korea
Source: J Clin Med. 2021 Dec 29;11(1):174. doi: 10.3390/jcm11010174 (PMC8745643; doi:10.3390/jcm11010174)

Figure S1. Probability of survival to discharge in validation cohort
(a) Probability of survival to discharge of the ED-PLANN score
(b) Probability of survival to discharge of the modified ED-PLANN score

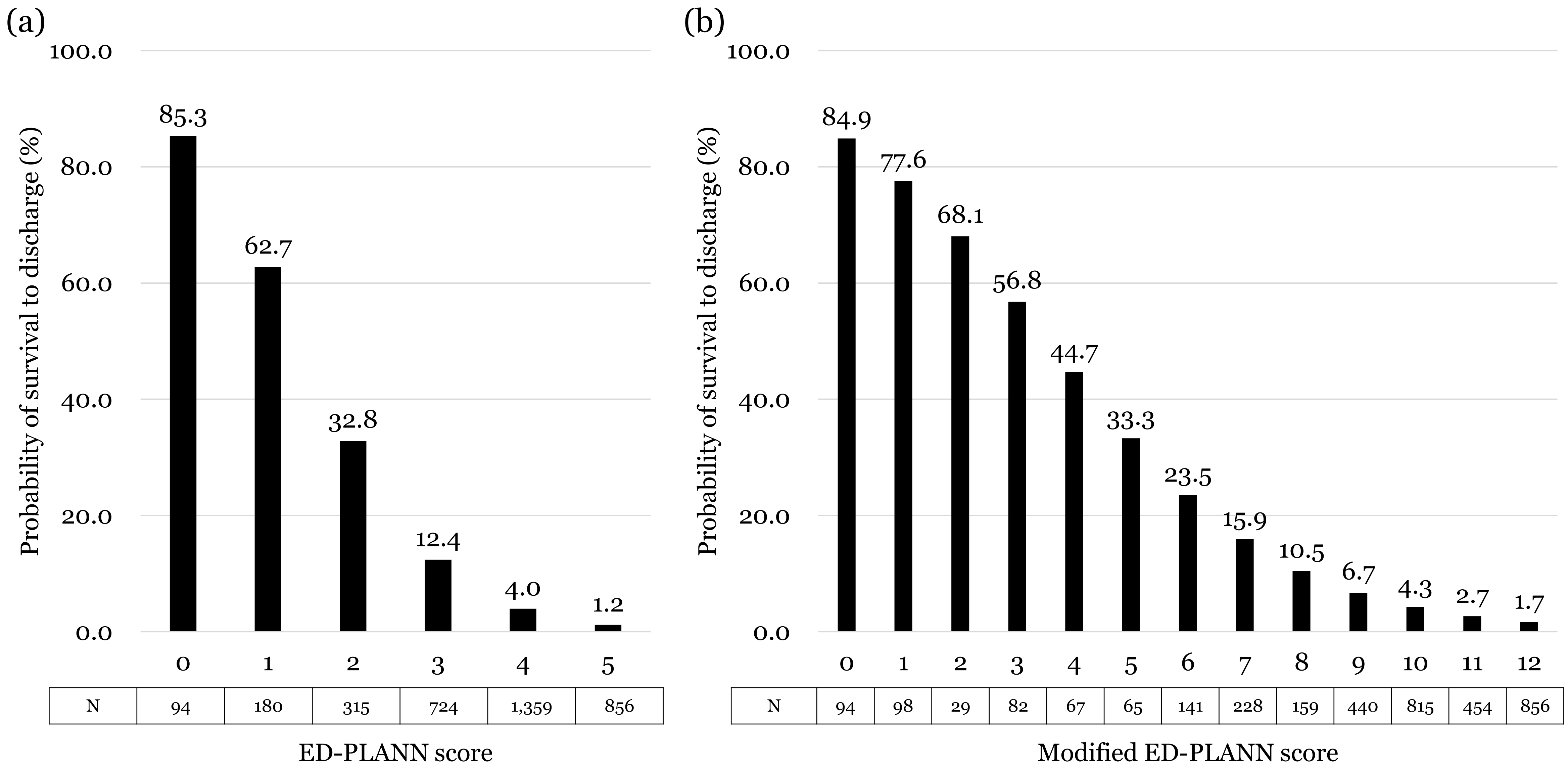

Supplement: Supplementary file 1 [file jcm-11-00174-s001.zip › JCM_Supplementary figure.pdf]
