# Supplementary material for: The ED-PLANN Score: A Simple Risk Stratification Tool for Out-of-Hospital Cardiac Arrests Derived from Emergency Departments in Korea
Source: J Clin Med. 2021 Dec 29;11(1):174. doi: 10.3390/jcm11010174 (PMC8745643; doi:10.3390/jcm11010174)
Supplement: Supplementary file 1 [file jcm-11-00174-s001.zip › JCM_Supplementary table final.pdf]

Supplementary

Table S1. Characteristics of excluded patients due to missing values for blood gas analysis.

	Total	Favorable neurological outcome	Poor neurological outcome	
	N (%)	N (%)	N (%)	p–value
**Total**	3,317	177	3,140	
Sex, female	2,076 (62.6)	143 (80.8)	1,933 (61.6)	<0.01
Age, year				<0.01
18–49	447 (13.5)	69 (39.0)	378 (12.0)	
50–59	508 (15.3)	47 (26.6)	461 (14.7)	
60–69	557 (16.8)	40 (22.6)	517 (16.5)	
70–79	882 (26.6)	17 (9.6)	865 (27.5)	
80–120	923 (27.8)	4 (2.3)	919 (29.3)	
Median (IQR)	72 (57-81)	54 (45-64)	73 (59-81)	<0.01
Past medical history				
Diabetes mellitus	788 (23.8)	24 (13.6)	764 (24.3)	<0.01
Hypertension	1,323 (39.9)	64 (36.2)	1,259 (40.1)	0.30
Dyslipidemia	155 (4.7)	11 (6.2)	144 (4.6)	0.32
Residence of patient				<0.01
Metropolitan	942 (28.4)	19 (10.7)	923 (29.4)	
Place of arrest				<0.01
Public	832 (25.1)	87 (49.2)	745 (23.7)	
Private	2,123 (64.0)	56 (31.6)	2,067 (65.8)	
Others	362 (10.9)	34 (19.2)	328 (10.4)	
Witnessed	1,836 (55.4)	154 (87.0)	1,682 (53.6)	<0.01
Bystander CPR	1,643 (49.5)	113 (63.8)	1,530 (48.7)	<0.01
Bystander defibrillation	20 (0.6)	5 (2.8)	15 (0.5)	<0.01
Initial shockable rhythm at the scene	525 (15.8)	135 (76.3)	390 (12.4)	<0.01
EMS time, minutes				
Response time interval, median (IQR)	7 (6–10)	7 (5–10)	8 (6–10)	<0.01
Scene time interval, median (IQR)	12 (8–18)	10 (6–13)	12 (8–19)	<0.01

Transport time interval, median (IQR)	9 (6–14)	11 (8–18)	9 (6–13)	<0.01
Prehospital treatment				
EMS defibrillation	768 (23.2)	143 (80.8)	625 (19.9)	<0.01
Mechanical CPR device	236 (7.1)	4 (2.3)	232 (7.4)	<0.01
Epinephrine	451 (13.6)	6 (3.4)	445 (14.2)	<0.01
Prehospital ROSC	298 (9.0)	153 (86.4)	145 (4.6)	<0.01
Post–resuscitation care				
TTM	158 (4.8)	42 (23.7)	116 (3.7)	<0.01
Reperfusion therapy	251 (7.6)	123 (69.5)	128 (4.1)	<0.01
ECMO	11 (0.3)	0 (0.0)	11 (0.4)	0.40
Survival to discharge	248 (7.5)	-	-	
Favorable neurological outcome	177 (5.3)	-	-	

Abbreviations: IQR, interquartile range; CPR, cardiopulmonary resuscitation; EMS, emergency medical services; ROSC, return of spontaneous circulation; TTM, targeted temperature management; ECMO, extracorporeal membrane oxygenation.

Table S2. Characteristics of external validation cohort population.

	Total	Favorable neurological outcome	Poor neurological outcome	
	N (%)	N (%)	N (%)	p–value
**Total**	3,528	289	3,239	
Sex, female	2,344 (66.4)	234 (81.0)	2,110 (65.1)	<0.01
Age, year				<0.01
18–49	494 (14.0)	105 (36.3)	389 (12.0)	
50–59	587 (16.6)	90 (31.1)	497 (15.3)	
60–69	663 (18.8)	58 (20.1)	605 (18.7)	
70–79	880 (24.9)	27 (9.3)	853 (26.3)	
80–120	904 (25.6)	9 (3.1)	895 (27.6)	
Median (IQR)	70 (57–80)	54 (47–63)	71 (58–80)	<0.01
Past medical history				
Diabetes mellitus	924 (26.2)	50 (17.3)	874 (27.0)	<0.01
Hypertension	1,397 (39.6)	100 (34.6)	1,297 (40.0)	0.04
Dyslipidemia	141 (4.0)	28 (9.7)	113 (3.5)	<0.01
Residence of patient				0.66
Metropolitan	992 (28.1)	78 (27.0)	914 (28.2)	
Place of arrest				<0.01
Public	848 (24.0)	114 (39.4)	734 (22.7)	
Private	2,143 (60.7)	112 (38.8)	2,031 (62.7)	
Others	537 (15.2)	63 (21.8)	474 (14.6)	
Witnessed	2,055 (58.2)	229 (79.2)	1,826 (56.4)	<0.01
Bystander CPR	1,754 (49.7)	179 (61.9)	1,575 (48.6)	<0.01
Bystander defibrillation	43 (1.2)	19 (6.6)	24 (0.7)	<0.01
Initial shockable rhythm at the scene	645 (18.3)	211 (73.0)	434 (13.4)	<0.01
EMS time, minutes				
Response time interval, median (IQR)	7.5 (6–10)	7 (5–9)	8 (6–10)	<0.01
Scene time interval, median (IQR)	13 (9–18)	10 (7–16)	13 (9–18)	<0.01
Transport time interval, median (IQR)	10 (6–14)	12 (7–17)	9 (6–13)	<0.01
Prehospital treatment				
EMS defibrillation	861 (24.4)	227 (78.5)	634 (19.6)	<0.01
Mechanical CPR device	316 (9.0)	6 (2.1)	310 (9.6)	<0.01
Epinephrine	542 (15.4)	19 (6.6)	523 (16.1)	<0.01
Prehospital ROSC	433 (12.3)	207 (71.6)	226 (7.0)	<0.01
Initial blood gas analysis				
pH, median (IQR)	6.93 (6.8.–7.09)	7.27 (7.16–7.34)	6.91 (6.80–7.04)	<0.01
pCO2, median (IQR)	75.8 (51.4–98.7)	37.9 (32.3–46.1)	78.7 (56.0–101.0)	<0.01

pO2, median (IQR)	44.0 (21.6–77.3)	87.4 (63.6–144.0)	40.5 (20.6–71.0)	<0.01
lactate, median (IQR)	13.7 (9.9–16.4)	5.3 (2.4–10.2)	14.6 (10.5–17.0)	<0.01
Post–resuscitation care				
TTM	301 (8.5)	85 (29.4)	216 (6.7)	<0.01
Reperfusion therapy	433 (12.3)	207 (71.6)	226 (7.0)	<0.01
ECMO	78 (2.2)	6 (2.1)	72 (2.2)	0.03
Survival to discharge	450 (12.8)	-	-	
Favorable neurological outcome	289 (8.2)	-	-	

Abbreviations: IQR, interquartile range; CPR, cardiopulmonary resuscitation; EMS, emergency medical services; ROSC, return of spontaneous circulation; TTM, targeted temperature management; ECMO, extracorporeal membrane oxygenation.

Table S3. Comparison of blood gas analysis components between before and after multiple imputation for the external validation cohort.

**Parameter**	Before imputation	After imputation	Imputation, N (%)
	Median (IQR)	Median (IQR)	
**pH**	6.93 (6.80–7.09)	6.93 (6.80–7.08)	246 (3.0)
pCO2, mmHg	72.1 (48.1–96.3)	76.7 (52.2–99.8)	255 (3.1)
pO2, mmHg	48.2 (23.3–82.7)	44.7 (21.0–79.5)	287 (3.5)
Lactate, mmol/L	11.8 (8.3–15.0)	13.6 (6.8–20.0)	3,263 (39.6)

Abbreviations: IQR, interquartile range.
